# Antibiotikaeinsatz: Psychologische Einflussfaktoren auf die Wahrnehmung und das Verhalten von Ärzt*innen und Patient*innen

**DOI:** 10.1007/s00103-026-04225-7

**Published:** 2026-04-01

**Authors:** Robert Böhm, Rian Gross, Lars Korn, Alina Schneider, Elisabeth D. C. Sievert, Cornelia Betsch

**Affiliations:** 1 Fakultät für Psychologie, Universität Wien, Universitätsstraße 7, 1010 Wien, Österreich; 2 Institut für Banken und Finanzen, Universität Innsbruck, Innsbruck, Österreich; 3 Gesundheitskommunikation, Implementationsforschung, Bernhard-Nocht-Institut für Tropenmedizin, Hamburg, Deutschland; 4 Gesundheitskommunikation, Institute for Planetary Health Behaviour, Universität Erfurt, Erfurt, Deutschland; 5 Patient-Reported Outcomes, Institut für Versorgungsforschung in der Dermatologie und den Pflegeberufen, Universitätsklinikum Hamburg-Eppendorf, Hamburg, Deutschland

**Keywords:** Antibiotikaresistenz, Antibiotic Stewardship, Antibiotikaeinsatz, Verhaltensänderung, Intervention, Antibiotic resistance, Antibiotic stewardship, Antibiotic use, Behavior change, Intervention

## Abstract

Antibiotikaresistenzen gefährden die Wirksamkeit standardisierter Therapien. Die Resistenzdynamik wird maßgeblich durch unsachgemäßen Antibiotikaeinsatz verstärkt. Dieser Beitrag stellt deshalb den Menschen in den Mittelpunkt der Bemühungen, den unsachgemäßen Antibiotikaeinsatz zu reduzieren, mit Fokus sowohl auf die ambulante als auch die stationäre Gesundheitsversorgung. Wir analysieren psychologische Faktoren, die zum einen die unsachgemäße Verschreibung von Antibiotika durch Ärzt*innen und zum anderen die unsachgemäße Anwendung von Antibiotika durch Patient*innen beeinflussen.

Auf Basis einer narrativen Übersicht werden folgende Faktoren aufseiten der Ärzt*innen identifiziert: (i) Wissen und Problembewusstsein, (ii) diagnostische und prognostische Unsicherheit, (iii) Prozesse beim Urteilen und Entscheiden, (iv) soziale Normen und Teamstrukturen sowie (v) Beziehungen zu Patient*innen. Aufseiten der Patient*innen erweisen sich (i) Wissen und Problembewusstsein, (ii) Prozesse beim Urteilen und Entscheiden und (iii) Umgang mit diagnostischer und prognostischer Unsicherheit als relevante Faktoren.

Zu jedem der Faktoren werden evidenzbasierte Maßnahmen vorgestellt, die sowohl die Verschreibungs- als auch die Einnahmepraxis von Antibiotika im Sinne einer verantwortungsvollen Einnahme positiv beeinflussen. Die systematische Integration solcher Ansätze in Antibiotic-Stewardship-Initiativen kann dabei helfen, die Verordnungs- und Einnahmepraxis von Antibiotika zu verbessern.

## Abstract (en)

Antibiotic resistance threatens the effectiveness of standard therapies. The dynamics of antibiotic resistance are substantially affected by inappropriate antibiotic use. Therefore, this article places humans at the center of efforts to reduce inappropriate antibiotic use, with a focus both on ambulatory and stationary care. We analyze psychological factors that contribute to inappropriate antibiotic prescribing by physicians and to inappropriate use by patients.

Drawing on a narrative review, we identify the following physician-side determinants: (i) knowledge and problem awareness, (ii) diagnostic and prognostic uncertainty, (iii) processes related to judgment and decision-making, (iv) social norms and team structures, and (v) relationships with patients. On the patient side, we identify (i) knowledge and problem awareness, (ii) processes related to judgment and decision-making, and (iii) dealing with diagnostic and prognostic uncertainty.

For each factor, we present evidence-based measures that can positively influence both appropriate prescribing and use. Systematically integrating these approaches into antibiotic stewardship initiatives can help improve prescribing and consumption practices.

## Hintergrund

Die stetige Zunahme von Antibiotikaresistenzen bedeutet für das Gesundheitswesen nicht nur längere Krankheitsverläufe und höhere Kosten, sondern auch eine zunehmende Gefährdung standardisierter Therapien, etwa in der Onkologie, Transplantationsmedizin oder Chirurgie [1]. Obwohl die Resistenzentwicklung von Bakterien ein natürlicher, evolutionsbiologischer Prozess ist, verstärken die unsachgemäße Verschreibung von Antibiotika durch Ärzt*innen und die unsachgemäße Anwendung durch Patient*innen in der ambulanten und stationären Gesundheitsversorgung diese Dynamik. Die zentrale Rolle von menschlichem Verhalten im Kontext von Antibiotikaresistenz und die Wichtigkeit, dieses Verhalten zu verändern, werden deshalb auch in der „One Health Priority Research Agenda for Antimicrobial Resistance“ der Quadripartite-Organisationen betont – also der Ernährungs- und Landwirtschaftsorganisation der Vereinten Nationen (FAO), dem Umweltprogramm der Vereinten Nationen (UNEP), der Weltgesundheitsorganisation (WHO) und der Weltorganisation für Tiergesundheit (WOAH; [2]).

In Deutschland gibt es bereits etablierte Antibiotic-Stewardship(ABS)-Initiativen, die darauf abzielen, den angemessenen Einsatz von Antibiotika zu fördern (siehe z. B. Deutsche Antibiotika Resistenzstrategie DART 2030). Diese bündeln Maßnahmen wie Leitlinien, Fort- und Weiterbildungen, Feedback zu Verordnungsraten, Point-of-Care-Diagnostik und Qualitätsindikatoren im ambulanten und stationären Bereich mit regionalen und klinikbasierten Initiativen. In der Praxis liegt der Fokus dieser Programme jedoch häufig auf klinischen Ergebnissen (z. B. Reduktion von unsachgemäßen Antibiotikaverordnungen) und den damit in Verbindung stehenden klinischen Prozessen (z. B. Implementierung von Diagnostik); seltener werden zugrunde liegende psychologische Einflussfaktoren des unsachgemäßen Antibiotikaeinsatzes adressiert. Psychologische Maßnahmen und Interventionen beschränken sich häufig auf die Vermittlung von Wissen, was zwar eine wichtige Grundlage darstellt, für das Erreichen nachhaltiger Verhaltensänderungen jedoch in der Regel nicht ausreicht und auch das Potenzial psychologisch basierter Interventionen nicht ausschöpft.

**Abb. 1:**
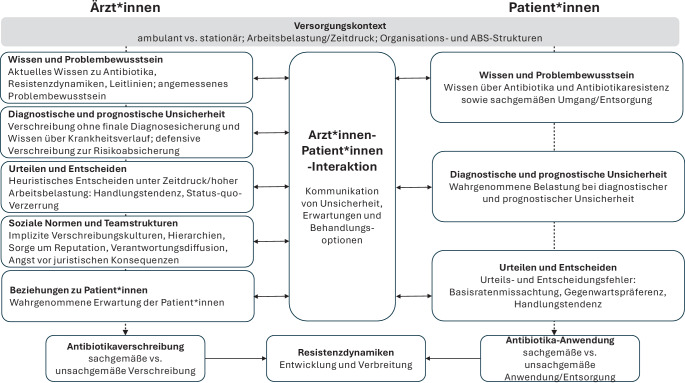
Psychologische Einflussfaktoren beim Antibiotikaeinsatz. Anmerkung. *ABS* Antibiotic Stewardship

**Abb. 2:**
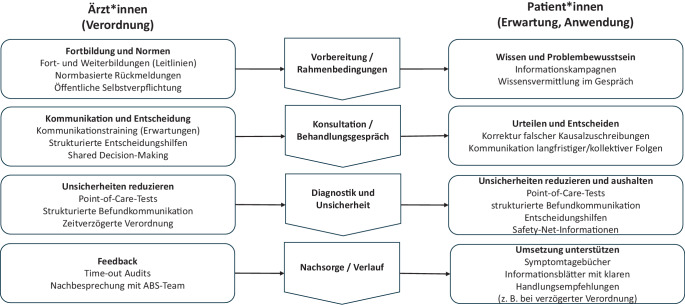
Evidenzbasierte Interventionen entlang des Entscheidungsprozesses beim Antibiotikaeinsatz. Anmerkung. *ABS* Antibiotic Stewardship

Ziel dieses Beitrags ist es, aktuelle Erkenntnisse zu psychologischen Einflussfaktoren darzustellen, die zu unsachgemäßer Antibiotikaverschreibung und -anwendung beitragen. Wie Abb. 1 verdeutlicht, werden in Ärzt*innen-Patient*innen-Interaktionen Entscheidungen über den Einsatz von Antibiotika von beiden Interaktionspartner*innen beeinflusst. Um diese Dynamiken verständlich zu machen, betrachten wir sie im Folgenden getrennt und entlang des klinischen Entscheidungsprozesses: Zunächst analysieren wir Faktoren, die Ärzt*innen zu unsachgemäßen Verschreibungen von Antibiotika verleiten. Anschließend richten wir den Fokus auf Patient*innen und die psychologischen Prozesse, die zu deren unsachgemäßer Nachfrage und Einnahme beitragen. Zudem präsentieren wir evidenzbasierte Interventionen, die diese Faktoren gezielt adressieren und dadurch unsachgemäße Verschreibung und Einnahme von Antibiotika reduzieren können (Abb. 2). Hierbei fokussieren wir bewusst nicht nur auf qualitative Studien zum Antibiotikagebrauch, sondern auch auf Untersuchungen, die psychologische Prozesse und damit verbundene Interventionen mittels Kausalinferenz (insbesondere in randomisierten Designs) analysieren. Der Beitrag liefert damit Impulse, bestehende ABS-Initiativen weiterzuentwickeln und zu stärken.

Der vorliegende Beitrag fokussiert auf die ambulante und stationäre Gesundheitsversorgung mit besonderem Blick auf den Kontext deutschsprachiger Länder (D, A, CH – DACH), in dem Antibiotika verschreibungspflichtig sind. Nicht Gegenstand dieses Beitrags sind andere relevante Sektoren, die zur Resistenzdynamik beitragen (z. B. Landwirtschaft, Veterinärmedizin, Abwasser/Umwelt), sowie Verhaltensweisen, die die Resistenzbildung indirekt beeinflussen, ohne unmittelbar die Verschreibung oder Einnahme zu betreffen (z. B. Impf- und Hygieneverhalten). Diese Eingrenzung dient der analytischen Schärfung: Im Mittelpunkt stehen psychologische Faktoren, die unmittelbar auf den Einsatz von Antibiotika wirken.

## Unsachgemäße Verschreibungen von Ärzt*innen

Die unsachgemäße Verschreibung von Antibiotika wird durch eine Vielzahl von Faktoren beeinflusst. Eine aktuelle Metasynthese zeigt, dass insbesondere kontextuelle (z. B. Ressourcen und Zeit) sowie soziale Faktoren (z. B. sozialer Einfluss und Normen) das Verschreibungsverhalten maßgeblich prägen [3]. Im Folgenden stellen wir die wichtigsten Einflussfaktoren vor und führen zu jedem der Faktoren evidenzbasierte Interventionen auf, um unsachgemäße Antibiotikaverordnungen zu reduzieren: (i) Wissen und Problembewusstsein, (ii) diagnostische und prognostische Unsicherheit, (iii) Urteilen und Entscheiden, (iv) soziale Normen und Teamstrukturen sowie (iv) Beziehungen zu Patient*innen.

### Wissen und Problembewusstsein von Ärzt*innen

Ein zentrales Merkmal verschreibender Ärzt*innen, das die Antibiotikaverordnung beeinflusst, ist neben der professionellen Rolle und den psychologischen Kompetenzen insbesondere ihr fachliches Wissen. Ausreichendes, aktuelles Wissen über Antibiotika, Antibiotikaresistenz, ABS-Konzepte sowie entsprechende Praxisleitlinien und eine regelmäßige Auffrischung dieser Expertise sind grundlegende Voraussetzungen für eine sachgemäße Verordnungspraxis. Insbesondere ein Problembewusstsein für Antibiotikaresistenz führt zu komplexeren und damit angemesseneren Verschreibungsentscheidungen [4]. Eine aktuelle Studie von Fal et al. [5] zeigt, dass Ärzt*innen in Deutschland insgesamt über ein gutes Wissen zu Antibiotika verfügen. Dennoch waren sich nur 23 % der Befragten der langfristigen Nebenwirkungen bewusst. Dies unterstreicht die Relevanz regelmäßiger Fort- und Weiterbildungen als potenzielle Ansatzpunkte für Interventionen.

Schulungsprogramme und Online-Trainings haben nachweislich positive und auch langfristige Effekte gezeigt, die sich in sinkenden Antibiotikaverschreibungsraten widerspiegeln [6]. In diesen Fortbildungsmaßnahmen werden nicht nur der aktuelle Stand der Forschung und die geltenden Behandlungsleitlinien vermittelt, sondern auch praxisorientierte Kommunikationsstrategien trainiert, um besser auf die Sorgen und Erwartungen der Patient*innen eingehen zu können. Darüber hinaus lernen die Teilnehmenden, wie interaktive Informationsmaterialien gezielt im Patient*innengespräch eingesetzt werden können, um den Dialog zu unterstützen und die gemeinsame Entscheidungsfindung zu fördern (siehe Sektionen „Beziehungen zu Patient*innen“ und „Urteilen und Entscheiden bei Patient*innen“).

### Diagnostische und prognostische Unsicherheit von Ärzt*innen

Über ausbildungsbezogene Merkmale der Ärzt*innen hinaus spielen diagnostische und prognostische Unsicherheit eine zentrale Rolle bei Entscheidungen über den Einsatz von Antibiotika. Behandlungsentscheidungen werden häufig getroffen, ohne dass eindeutig feststeht, ob den von Patienten*innen berichteten Symptomen eine bakterielle Infektion zugrunde liegt (diagnostische Unsicherheit). Selbst wenn eine bakterielle Ätiologie wahrscheinlich oder sicher ist, bleibt der weitere Krankheitsverlauf oft ungewiss (prognostische Unsicherheit). Diese Unsicherheiten erhöhen die Wahrscheinlichkeit, eine Antibiotikatherapie zu verschreiben oder fortzuführen [7], da die Sorge besteht, relevante Erreger zu übersehen, oder um potenziell negative Konsequenzen schwerer Krankheitsverläufe zu vermeiden („better safe than sorry“).

Zur Verringerung diagnostischer Unsicherheiten können Point-of-Care-Tests (z. B. C‑reaktives-Protein-Schnelltest, Streptokokken-Schnelltest) eingesetzt werden. Schulungen zur korrekten Anwendung sowie der regelmäßige Einsatz dieser Tests insbesondere im ambulanten Kontext haben sich als wirksam erwiesen, um unnötige Antibiotikaverschreibungen zu reduzieren, ohne die Genesung oder Zufriedenheit der Patient*innen zu beeinträchtigen [8]. Die größten Effekte zeigen sich, wenn diese Maßnahmen mit Kommunikationstrainings für das Patient*innengespräch kombiniert werden [9].

Ein weiterer evidenzbasierter Ansatz zur Reduktion der Verschreibungsraten angesichts diagnostischer und prognostischer Unsicherheit ist die zeitverzögerte Verordnung (*Delayed Prescription; *[10]). Dabei wird ambulanten Patient*innen, bei denen kein sofortiger Antibiotikaeinsatz erforderlich ist, zunächst empfohlen, einige Tage abzuwarten, um den weiteren Krankheitsverlauf zu beobachten (*Watchful Waiting*). Das Rezept wird entweder auf ein späteres Datum ausgestellt oder soll erst eingelöst werden, wenn die Beschwerden fortbestehen oder sich verschlimmern und dabei vorher festgelegten Kriterien entsprechen. Diese Strategie wird unter anderem im Vereinigten Königreich eingesetzt [11], wobei sie oftmals zur Reduktion der Verschreibungsraten beiträgt. Entscheidend für den Erfolg sind klare Handlungsanweisungen für die Patient*innen, einschließlich Empfehlungen zur symptomorientierten Behandlung und eindeutiger Kriterien für eine erneute ärztliche Vorstellung [12].

### Urteilen und Entscheiden bei Ärzt*innen

Das Arbeitsumfeld beeinflusst Wahrnehmung, Aufmerksamkeit, Gedächtnis und Entscheidungsprozesse von Ärzt*innen [3]. Unter hoher Arbeitsbelastung und Zeitdruck greifen Ärzt*innen häufiger auf Heuristiken zurück, die Entscheidungsprozesse beschleunigen, aber auch die Wahrscheinlichkeit von Fehlverschreibungen steigern können. Im Kontext von Antibiotikaverschreibungen sind 2 kognitive Verzerrungen zentral: die Handlungstendenz (*Action Bias*) und die Status-quo-Verzerrung (*Status Quo Bias*).

Die Handlungstendenz beschreibt die Neigung, aktiv zu handeln, statt eine Handlung zu unterlassen, selbst wenn die Handlung potenziell zu schlechteren Ergebnissen führt. Die Handlungstendenz nimmt bei negativen Erfahrungen im Fall von unterlassenen Handlungen zu (z. B. eine in der Vergangenheit unbehandelte Krankheit, die zu schweren Symptomen führte). Eine unterlassene Behandlung durch Ärzt*innen kann von Patient*innen zudem als Untätigkeit oder sogar Inkompetenz interpretiert werden, während eine Handlung eher Engagement signalisiert [13].

Die Status-quo-Verzerrung beschreibt die Tendenz zur Bewahrung des Status quo und wird oft mit Entscheidungsträgheit in Zusammenhang gebracht, d. h. mit der Tendenz, Entscheidungen zu bevorzugen, die mit geringerem kognitiven Aufwand verbunden sind. Zusätzliche Diagnostik und zeitverzögerte Verordnungen gehen mit einem gewissen Zeit- und Organisationsaufwand einher. Die Beibehaltung des Status quo (einmalige und sofortige Antibiotikaverordnung) kann damit als kognitiv erleichternd angesehen werden [14].

Eine wirksame Strategie zur Reduktion kognitiver Belastungen und zur Förderung strukturierter Entscheidungen ist der Einsatz von Entscheidungshilfen. Diese vorzugsweise evidenzbasierten Instrumente stellen aktuelle Leitlinien, Behandlungsoptionen und Alternativen übersichtlich dar [15]. Sie können sowohl die individuelle Entscheidungsfindung von Ärzt*innen als auch die gemeinsame Entscheidungsfindung mit Patient*innen unterstützen. Eingebettet in das Behandlungsgespräch fördern sie Transparenz und partizipative Kommunikation (siehe Sektion „Beziehungen zu Patient*innen“). Eine aktuelle Metaanalyse zeigt, dass digitale oder gedruckte Entscheidungshilfen unnötige Antibiotikaverschreibungen reduzieren [16]. Zudem verbessert ihre Implementierung nachweislich die Einhaltung von Leitlinien und trägt damit zu einem sachgemäßen Antibiotikaeinsatz bei [17].

### Soziale Normen und Teamstrukturen

Soziale Normen innerhalb von Teams und beruflichen Beziehungen sind ein wesentlicher Faktor für das Verschreibungsverhalten von Ärzt*innen, insbesondere im stationären Kontext [3]. Die Internalisierung kollegialer Praktiken sowie informelle Verschreibungsetiketten und regionale Verordnungskulturen – also ungeschriebene Regeln des Verordnungsverhaltens – können dabei maßgeblich wirken [18]. Auch Reputationsaspekte spielen eine Rolle: Die Sorge, als zu zurückhaltend oder nachlässig bei den Verschreibungen wahrgenommen zu werden, beeinflusst die Entscheidungen ebenso [19] wie Verantwortungsdiffusion und die Angst vor möglichen Rechtsfolgen [20]. Weiterhin prägen Hierarchien im Team die sozialen Dynamiken im klinischen Kontext und spielen eine zentrale Rolle im Verschreibungsverhalten [3]. Besonders jüngere oder sich in Ausbildung befindende Ärzt*innen orientieren sich häufig an den Gepflogenheiten und Entscheidungen ihrer Vorgesetzten, wodurch sich bestimmte Verschreibungskulturen etablieren. Im klinischen Alltag wird zudem oft vermieden, Kolleg*innen zu widersprechen, was zu einer Kultur der Zurückhaltung oder gar Nichteinmischung führen kann [18]. Feedback oder Kritik wird häufig aus Respekt vor Hierarchien oder zur Wahrung kollegialer Beziehungen unterlassen, was solche Strukturen weiter festigt.

Zur Etablierung förderlicher sozialer Normen zur sachgemäßen Verschreibung von Antibiotika haben sich verschiedene Ansätze bewährt. Ein wirkungsvolles Instrument sind regelmäßige Rückmeldungen zum individuellen Verschreibungsverhalten im Vergleich zu Kolleg*innen (deskriptive Norm) oder zu geltenden Verschreibungsrichtlinien (injunktive Norm; insbesondere, wenn diese der Verschreibungskultur widerspricht). Besonders effektiv sowohl im ambulanten als auch im stationären Kontext ist die Gegenüberstellung der eigenen Verordnungsrate mit den besten Vergleichswerten von Kolleg*innen – also den niedrigsten Verschreibungsraten in der gleichen Klinik oder in anderen Kliniken und Praxen bei gleichen Diagnosen – oder eine gezielte Rückmeldung an Personen mit überdurchschnittlich vielen Antibiotikaverordnungen [21]. Im stationären Kontext können darüber hinaus die vorher erwähnten Entscheidungshilfen insbesondere jüngeren Kolleg*innen dabei helfen, ihre Verschreibungsentscheidungen gegenüber Vorgesetzten zu rechtfertigen.

Eine Variante der injunktiven Normkommunikation im stationären Kontext sind sogenannte Time-out-Audits. Dabei werden regelmäßig – etwa 2‑mal wöchentlich – alle laufenden Antibiotikatherapien anhand standardisierter Checklisten überprüft und gegebenenfalls angepasst. Die Ergebnisse und Verschreibungsdaten werden monatlich zusammengefasst und dem Behandlungsteam zurückgemeldet. Evaluationen zeigen, dass Time-out-Audits zu einem gezielteren Antibiotikaeinsatz und zu geringeren Behandlungskosten führen, auch wenn die Gesamtmenge verordneter Antibiotika häufig unverändert bleibt [22]. Förderlich für eine erfolgreiche Umsetzung sind Schulungen zur Anwendung der Checklisten sowie die Begleitung durch ein ABS-Team [23]. Darüber hinaus unterstützen diese Audits durch ihre strukturierte Herangehensweise kontinuierliches Lernen und den nachhaltigen, rationalen Einsatz von Antibiotika im Klinikalltag.

Ein weiterer Erfolg versprechender Ansatz sowohl für den ambulanten als auch für den stationären Kontext ist die öffentliche Selbstverpflichtung zur Reduktion unangemessener Antibiotikaverschreibungen und zum Erhalt der Wirksamkeit von Antibiotika. Meeker und Kolleg*innen zeigten, dass solche Selbstverpflichtungserklärungen die Anzahl unnötiger Verschreibungen senken, ohne dabei angemessene Verordnungen zu beeinträchtigen [24]. In der Praxis kann dies durch Aushänge erfolgen, auf denen Ärzt*innen ihre Absicht mit Unterschrift und Foto veröffentlichen. Dadurch wird die Selbstverpflichtung an Patient*innen und Kolleg*innen kommuniziert, was die Einhaltung der sozialen Norm fördert.

### Beziehungen zu Patient*innen

Auch Dynamiken in der Interaktion zwischen Ärzt*innen und Patient*innen beeinflussen die Verschreibungspraxis erheblich [3]. Besonders die ärztliche Wahrnehmung, Patient*innen erwarteten oder forderten Antibiotika, ist in Studien ein konsistenter und teils starker Prädiktor für Verschreibungen (häufig stärker als die von Patient*innen berichtete Erwartung), wobei die Richtung und Stärke kontextabhängig variieren [25]. Die indirekten Erwartungseffekte treten selbst dann auf, wenn die Ärzt*innen eine bakterielle Infektion als unwahrscheinlich einschätzen [26]. Hinzu kommen Verantwortungsgefühle und die Wertschätzung einer positiven Beziehung mit den Patient*innen, die das Verschreibungsverhalten zusätzlich beeinflussen können [19].

Das Gespräch zwischen Ärzt*in und Patient*in bildet die zentrale Schnittstelle, an der Ärzt*innen aktiv zu einer rationalen Entscheidungsfindung beitragen können. Im Rahmen der gemeinsamen Entscheidungsfindung (*Shared Decision-Making*; [27]) ermöglichen evidenzbasierte Entscheidungshilfen und Informationsmaterialien, gezielt auf Sorgen, Erwartungen und Informationsbedürfnisse der Patient*innen einzugehen. Ebenso kann durch die Anwendung solcher Instrumente die Verschreibungsentscheidung gegenüber Kolleg*innen gerechtfertigt werden, selbst wenn sie der vorherrschenden Verschreibungskultur widerspricht. Eine Beobachtungsstudie konnte zeigen, dass Ärzt*innen mögliche Nebenwirkungen oder das Thema Antibiotikaresistenz nur selten ansprechen; wird hingegen Shared Decision-Making praktiziert, werden Nutzen und Risiken einer Antibiotikatherapie häufiger und ausführlicher thematisiert [28]. Der Einsatz entsprechender Instrumente führt nachweislich zu einer Verringerung unsachgemäßer Verschreibungen [29].

## Unsachgemäße Antibiotikaeinnahme von Patient*innen

Während lange Zeit Ärzt*innen sowie deren Verschreibungspraxis im Mittelpunkt der Diskussion über Antibiotikaresistenz standen, zeigt sich zunehmend, dass auch Patient*innen wesentlich zur Entstehung und Verbreitung resistenter Erreger beitragen. Wie bereits erwähnt, können sie ärztliche Entscheidungen durch ihre Erwartungen indirekt beeinflussen (siehe Sektion „Beziehungen zu Patient*innen“). Darüber hinaus wirken sie durch ihr Einnahmeverhalten unmittelbar auf Resistenzdynamiken ein – etwa durch das Aufbewahren von Restbeständen oder die Weitergabe von Antibiotika [30]. Patient*innen stellen somit eine zentrale Zielgruppe für verhaltensbezogene Interventionen dar. Im Folgenden beschreiben wir die wichtigsten psychologischen und verhaltensbezogenen Mechanismen, über die Patient*innen Antibiotikaresistenzen beeinflussen, sowie evidenzbasierte Ansätze, die folgende Prozesse gezielt adressieren: (i) Wissen und Problembewusstsein, (ii) Urteilen und Entscheiden sowie (iii) Umgang mit diagnostischer und prognostischer Unsicherheit.

### Wissen und Problembewusstsein von Patient*innen

Zahlreiche Studien belegen weitverbreitete Fehlannahmen in der Bevölkerung: Viele Menschen haben unzureichendes Wissen über die Ätiologie von Infektionskrankheiten und gehen beispielsweise davon aus, dass Erkältungen oder grippale Infekte bakteriell bedingt sind und sich mit Antibiotika behandeln lassen. In einer aktuellen Umfrage aus Deutschland hielten 17 % der Befragten Antibiotika für wirksam gegen Virusinfektionen [31]. Selbst wenn Patient*innen schon von Antibiotikaresistenzen gehört haben, bleibt das Verständnis häufig oberflächlich. Viele gehen fälschlich davon aus, dass der menschliche Körper resistent wird – nicht die Bakterien [32]. Außerdem haben Patient*innen unzureichendes Wissen darüber, wie Antibiotikareste aus früheren Therapien fachgerecht entsorgt oder zurückgegeben werden können [33], was zur Aufbewahrung und damit potenziell zur unsachgemäßen Selbstmedikation bei zukünftigen Infektionen führen kann.

Es bleibt deshalb ein zentrales Ziel von Informationskampagnen und Gesprächen zwischen Ärzt*innen und Patient*innen, auf die Unwirksamkeit von Antibiotika bei viralen Erkrankungen hinzuweisen und Regeln für den Umgang mit und die Entsorgung von Antibiotika zu vermitteln. Aufklärung und Wissensvermittlung können die Erwartung von Patient*innen, ein Rezept für ein Antibiotikum zu erhalten, signifikant senken [34].

### Umgang mit diagnostischer und prognostischer Unsicherheit

Viele Patient*innen empfinden diagnostische und prognostische Unsicherheit als belastend und bevorzugen daher die vermeintlich „sichere“ Option eines Antibiotikums [35]. Diese Tendenz ist ausgeprägter, wenn in der Vergangenheit positive Erfahrungen mit Antibiotika gemacht wurden [36]. Vor diesem Hintergrund gewinnt die ärztliche Kommunikation an Bedeutung: Neuere experimentelle Befunde zeigen, dass eine offene Ansprache diagnostischer Unsicherheit Antibiotikaerwartungen nicht erhöht, sondern senken kann – vorausgesetzt, sie wird mit klaren Handlungsoptionen kombiniert. In einer Studie von Sievert et al. [37] reduzierten Ärzt*innen, die diagnostische oder prognostische Unsicherheit explizit benannten und zugleich eine zeitverzögerte Verordnung anboten, die Erwartung einer Antibiotikaverordnung bei Patient*innen signifikant, ohne das Vertrauen der Patient*innen zu beeinträchtigen. Diese Ergebnisse verdeutlichen, dass diagnostische Unsicherheit konstruktiv genutzt werden kann, um eine sachgemäße Antibiotikaeinnahme zu fördern.

Diagnostische Unsicherheit lässt sich zudem durch Point-of-Care-Tests (siehe Sektion „Diagnostische und prognostische Unsicherheit von Ärzt*innen“) und strukturierte Befundkommunikation verringern. Wenn die Testergebnisse gemeinsam besprochen und visuell dargestellt werden, verstehen Patient*innen besser, warum ein Antibiotikum nicht erforderlich ist [34]. Ergänzend helfen sogenannte Safety-Net-Informationen, also klare Hinweise, wann eine Wiedervorstellung sinnvoll ist [38]. So wird diagnostische Unsicherheit transparent kommuniziert, ohne das Vertrauen in die Behandlungsqualität zu untergraben.

### Urteilen und Entscheiden bei Patient*innen

Urteils- und Entscheidungsfehler können auch bei Patient*innen zur unsachgemäßen Anwendung von Antibiotika beitragen. Hier stellen wir 3 solcher Urteils- und Entscheidungsfehler und mögliche Interventionsstrategien vor, die im Kontext von unsachgemäßem Antibiotikagebrauch von Patient*innen untersucht wurden: Basisratenmissachtung (*Base Rate Neglect*), Gegenwartspräferenz (*Present Bias*) und Handlungstendenz (*Action Bias*).

Verschwinden Symptome kurz nach der Antibiotikaeinnahme, wird die Heilung häufig fälschlich dem Antibiotikum zugeschrieben, auch wenn es sich um eine Spontanremission handelt [39]. Dies hat zur Folge, dass die zukünftige Nachfrage steigt [40]. Solche Fehlschlüsse lassen sich durch gezielte Informationen zu Basisraten von selbstlimitierenden Infektionen korrigieren [39]: Werden Patient*innen im Rahmen einer zeitverzögerten Verordnung (*Delayed Prescription*; siehe Sektionen zu „Umgang mit diagnostischer und prognostischer Unsicherheit“) darüber informiert, dass eine Vielzahl von Infekten auch ohne Antibiotikum abklingt, schreiben sie eine Genesung seltener dem Medikament zu [39]. Diese Befunde zeigen, dass durch die Korrektur falscher Kausalzuschreibungen die Basisratenmissachtung reduziert und damit die zukünftige Nachfrage nach Antibiotika gesenkt werden kann.

Ein weiterer Urteilsfehler, der zur unsachgemäßen Einnahme von Antibiotika beitragen kann, ist die Gegenwartspräferenz. Hierbei werden kurzfristige persönliche Vorteile – etwa eine rasche Genesung – stärker gewichtet als langfristige persönliche und gesellschaftliche Kosten und Risiken wie die Resistenzbildung [41]. Die Sichtbarkeit des eigenen Handelns und dessen Auswirkungen auf andere kann den Fokus vom kurzfristigen Eigennutz auf den langfristigen gesellschaftlichen Nutzen verschieben: In einem experimentellen interaktiven Versuchsaufbau zeigte sich, dass unsachgemäßer Antibiotikagebrauch vor allem bei anonymen Entscheidungen auftritt, bei denen die eigene Einnahme für andere nicht sichtbar ist. Wurden der Antibiotikagebrauch und dessen gesellschaftliche Auswirkungen sichtbar gemacht, reduzierte sich die unsachgemäße Antibiotikaeinnahme [42]. Rückmeldungen zum eigenen und zum Einnahmeverhalten anderer können somit den verantwortungsbewussten Antibiotikagebrauch fördern.

Eine weitere Studie konnte zeigen, dass das Hervorrufen von Empathie gegenüber zukünftigen Generationen – die in besonderem Ausmaß unter einer zunehmenden Antibiotikaresistenz leiden würden – den verantwortungsvollen Umgang mit Antibiotika fördert [43]. Erlebbare und immersive Formate, wie Simulationen in virtueller Realität oder Kurzfilme, die die Folgen individueller Übereinnahme von Antibiotika oder eine postantibiotische Zukunft (d. h. eine apokalyptische Zukunft, in der Antibiotika nicht mehr wirksam sind) veranschaulichen, erhöhen zudem das Problembewusstsein und tragen damit zur Reduktion der Gegenwartspräferenz bei [44, 45].

Viele Patient*innen empfinden passives Abwarten über die weitere Symptomentwicklung als unzufriedenstellend und bevorzugen aktives Handeln – ein Muster, das der sogenannten Handlungstendenz entspricht. Selbst wenn auf die Unwirksamkeit oder die potenziellen Risiken von Antibiotika hingewiesen wurde, wünschten sich rund 10 % der Befragten einer Studie aus dem Vereinigten Königreich dennoch eine Antibiotikaverschreibung [46]. Interventionen, die Abwarten als aktive Strategie legitimieren, können hier ansetzen. In einer experimentellen Studie führte die Einführung eines Symptomtagebuchs, in dem Patient*innen ihren Krankheitsverlauf dokumentierten, zu einer geringeren Nachfrage nach Antibiotika [47]. Ähnliche Effekte zeigte eine Untersuchung, in der Patient*innen während einer hypothetischen Konsultation ein von der Weltgesundheitsorganisation entwickeltes Informationsblatt [48] erhielten, das evidenzbasierte Fakten über Antibiotikaresistenz mit konkreten Handlungsoptionen kombinierte. Diese Intervention erhöhte die Akzeptanz einer zeitverzögerten Verordnung und verringerte die wahrgenommene Notwendigkeit einer sofortigen Antibiotikatherapie [49].

## Fazit und Ausblick

Dieser Beitrag bündelt etablierte und aktuelle Befunde zu psychologischen Faktoren, die unangemessene Antibiotikaverschreibungen durch Ärzt*innen und unsachgemäße Anwendungen durch Patient*innen begünstigen. Die Analyse unterstreicht die Bedeutung von Wissen und Problembewusstsein über Antibiotikaresistenz in beiden Gruppen. Allerdings genügt dies allein nicht, um den unsachgemäßen Antibiotikagebrauch deutlich zu verringern. Daher sollten Diagnostik, Verschreibungspraxis und die Kommunikation zwischen Ärzt*innen und Patient*innen so gestaltet werden, dass Urteils- und Entscheidungsfehler reduziert und Vertrauen erhalten oder gestärkt wird.

Keine der genannten Interventionen allein kann die zunehmende Antibiotikaresistenz stoppen und jede Intervention kann unsachgemäß angewandt oder potenziell missbraucht werden – z. B. indem Einnahmeentscheidungen an Patient*innen delegiert werden. Umso wichtiger ist ein geschulter Umgang. Da der ärztliche Alltag bereits stark belastet ist, braucht es gezielte Unterstützung durch klare Handlungsempfehlungen, evidenzbasierte Entscheidungshilfen und strukturierte Fortbildungen zur Kommunikation über Antibiotikaresistenz und ihre Folgen.

Über die Identifikation wirksamer Interventionen hinaus stellt sich für den Transfer in die Regelversorgung die Implementierungsfrage. Für die Verstetigung psychologischer Interventionen in ABS-Programmen sind daher (i) standardisierte Interventionsbausteine mit geeigneten Erfolgsindikatoren, (ii) wissenschaftlich begleitete Implementierung mit Feedbackschleifen und ggf. Deimplementierung, (iii) dauerhafte Monitoring- und Dateninfrastrukturen (inkl. standardisierter Schnittstellen) sowie (iv) ausreichende Ressourcen (Zeit, Qualifizierung, Brückenpersonal und nachhaltige Finanzierung) erforderlich.

Auch wenn einige der in unserem Beitrag dargestellten aktuellen Forschungsergebnisse bislang nur aus anderen Ländern vorliegen, sehen wir ein großes Potenzial für die Übertragung und Kontextualisierung der Befunde im DACH-Kontext. Durch die systematische Integration aktueller Erkenntnisse aus der Psychologie sowie den Sozial- und Verhaltenswissenschaften könnten ABS-Initiativen gezielt weiterentwickelt und optimiert werden.

## References

[1] World Health Organization (2023) Antimicrobial Resistance.

[2] World Health Organization, UNEP United Nations Environment Programme, World Organisation for Animal Health (2023) A one health priority research agenda for antimicrobial resistance.

[3] Mabaya G, Evans JM, Longo CJ, Morris AM (2025) A Behavioral Analysis of Factors That Influence Antibiotic Prescribing in Hospitals: A Metasynthesis of Reviews. Open Forum Infect Dis 12:ofae728. 10.1093/ofid/ofae728 39781373 10.1093/ofid/ofae728 PMC11707608

[4] Krockow EM, Colman AM, Chattoe-Brown E et al (2019) Balancing the risks to individual and society: a systematic review and synthesis of qualitative research on antibiotic prescribing behaviour in hospitals. J Hosp Infect 101:428–439. 10.1016/j.jhin.2018.08.007 30099092 10.1016/j.jhin.2018.08.007

[5] Fal AM, Stelzmüller I, Kardos P et al (2024) Antibiotics usage and avoidance in Germany and Poland: attitudes and knowledge of patients, physicians, and pharmacists. Antibiotics 13:1188. 10.3390/antibiotics13121188 39766578 10.3390/antibiotics13121188 PMC11672592

[6] Butler CC, Simpson SA, Dunstan F et al (2012) Effectiveness of multifaceted educational programme to reduce antibiotic dispensing in primary care: practice based randomised controlled trial. BMJ 344:d8173. 10.1136/bmj.d8173 22302780 10.1136/bmj.d8173 PMC3270575

[7] Pandolfo AM, Horne R, Jani Y et al (2022) Understanding decisions about antibiotic prescribing in ICU: an application of the Necessity Concerns Framework. BMJ Qual Saf 31:199–210. 10.1136/bmjqs-2020-012479 34099497 10.1136/bmjqs-2020-012479 PMC8899486

[8] Cals JWL, Butler CC, Hopstaken RM et al (2009) Effect of point of care testing for C reactive protein and training in communication skills on antibiotic use in lower respiratory tract infections: cluster randomised trial. BMJ 338:b1374. 10.1136/bmj.b1374 19416992 10.1136/bmj.b1374 PMC2677640

[9] Little P, Stuart B, Francis N et al (2013) Effects of internet-based training on antibiotic prescribing rates for acute respiratory-tract infections: a multinational, cluster, randomised, factorial, controlled trial. The Lancet 382:1175–1182. 10.1016/S0140-6736(13)60994-0 10.1016/S0140-6736(13)60994-0 PMC3807804 23915885

[10] Little P, Moore M, Kelly J et al (2014) Delayed antibiotic prescribing strategies for respiratory tract infections in primary care: pragmatic, factorial, randomised controlled trial. BMJ. 10.1136/BMJ.G1606 24603565 10.1136/bmj.g1606 PMC3944682

[11] Little P, Stuart B, Smith S et al (2017) Antibiotic prescription strategies and adverse outcome for uncomplicated lower respiratory tract infections: prospective cough complication cohort (3C) study. BMJ 357:j2148. 10.1136/bmj.j2148 28533265 10.1136/bmj.j2148 PMC5439222

[12] Spurling GK, Del Mar CB, Dooley L et al (2017) Delayed antibiotic prescriptions for respiratory infections. Cochrane Database Syst Rev. 10.1002/14651858.CD004417.pub5 28881007 10.1002/14651858.CD004417.pub5 PMC6372405

[13] Kiderman A, Ilan U, Gur I et al (2013) Unexplained complaints in primary care: evidence of action bias. J Fam Pract 62:408–413 24143333

[14] Wang SY, Cantarelli P, Groene O et al (2023) Patient expectations do matter - Experimental evidence on antibiotic prescribing decisions among hospital-based physicians. Health Policy 128:11–17. 10.1016/j.healthpol.2022.11.009 36450627 10.1016/j.healthpol.2022.11.009

[15] Gulliford MC, Prevost AT, Charlton J et al (2019) Effectiveness and safety of electronically delivered prescribing feedback and decision support on antibiotic use for respiratory illness in primary care: REDUCE cluster randomised trial. BMJ 364:l236. 10.1136/bmj.l236 30755451 10.1136/bmj.l236 PMC6371944

[16] Xu R, Wu L, Wu L et al (2023) Effectiveness of decision support tools on reducing antibiotic use for respiratory tract infections: a systematic review and meta-analysis. Front Pharmacol. 10.3389/fphar.2023.1253520 37745052 10.3389/fphar.2023.1253520 PMC10512864

[17] Rittmann B, Stevens MP (2019) Clinical Decision Support Systems and Their Role in Antibiotic Stewardship: a Systematic Review. Curr Infect Dis Rep 21:29. 10.1007/s11908-019-0683-8 31342180 10.1007/s11908-019-0683-8

[18] Charani E, Castro-Sanchez E, Sevdalis N et al (2013) Understanding the determinants of antimicrobial prescribing within hospitals: the role of „prescribing etiquette“. Clin Infect Dis Off Publ Infect Dis Soc Am 57:188–196. 10.1093/cid/cit212 10.1093/cid/cit212 PMC3689346 23572483

[19] Broom A, Broom J, Kirby E (2014) Cultures of resistance? A Bourdieusian analysis of doctors’ antibiotic prescribing. Soc Sci Med 110:81–88. 10.1016/j.socscimed.2014.03.030 24727665 10.1016/j.socscimed.2014.03.030

[20] Livorsi D, Comer A, Matthias MS et al (2015) Factors Influencing Antibiotic-Prescribing Decisions Among Inpatient Physicians: A Qualitative Investigation. Infect Control Hosp Epidemiol 36:1065–1072. 10.1017/ice.2015.136 26078017 10.1017/ice.2015.136 PMC4797059

[21] Raban MZ, Gonzalez G, Nguyen AD et al (2023) Nudge interventions to reduce unnecessary antibiotic prescribing in primary care: a systematic review. BMJ Open 13:e062688. 10.1136/bmjopen-2022-062688 36657758 10.1136/bmjopen-2022-062688 PMC9853249

[22] Lee TC, Frenette C, Jayaraman D et al (2014) Antibiotic Self-stewardship: Trainee-Led Structured Antibiotic Time-outs to Improve Antimicrobial Use. Ann Intern Med 161:S53–S58. 10.7326/M13-3016 25402404 10.7326/M13-3016

[23] Thom KA, Tamma PD, Harris AD et al (2019) Impact of a Prescriber-driven Antibiotic Time-out on Antibiotic Use in Hospitalized Patients. Clin Infect Dis 68:1581–1584. 10.1093/cid/ciy852 30517592 10.1093/cid/ciy852

[24] Meeker D, Knight TK, Friedberg MW et al (2014) Nudging Guideline-Concordant Antibiotic Prescribing: A Randomized Clinical Trial. JAMA Intern Med 174:425–431. 10.1001/jamainternmed.2013.14191 24474434 10.1001/jamainternmed.2013.14191 PMC4648560

[25] McKay R, Mah A, Law MR et al (2016) Systematic Review of Factors Associated with Antibiotic Prescribing for Respiratory Tract Infections. Antimicrob Agents Chemother 60:4106–4118. 10.1128/AAC.00209-16 27139474 10.1128/AAC.00209-16 PMC4914667

[26] Sirota M, Round T, Samaranayaka S, Kostopoulou O (2017) Expectations for antibiotics increase their prescribing: Causal evidence about localized impact. Health Psychol 36:402–409. 10.1037/hea0000456 28206788 10.1037/hea0000456

[27] Elwyn G, Frosch D, Thomson R et al (2012) Shared Decision Making: A Model for Clinical Practice. J Gen Intern Med 27:1361–1367. 10.1007/s11606-012-2077-6 22618581 10.1007/s11606-012-2077-6 PMC3445676

[28] Bakhit M, Del Mar C, Gibson E, Hoffmann T (2018) Shared decision making and antibiotic benefit-harm conversations: an observational study of consultations between general practitioners and patients with acute respiratory infections. BMC Fam Pract 19:165. 10.1186/s12875-018-0854-y 30292242 10.1186/s12875-018-0854-y PMC6173855

[29] Coxeter P, Mar CBD, McGregor L et al. (2015) Interventions to facilitate shared decision making to address antibiotic use for acute respiratory infections in primary care - Coxeter, P - 2015 | Cochrane Library. 10.1002/14651858.CD010907.pub2 PMC6464273 26560888

[30] McNulty CAM, Collin SM, Cooper E et al (2019) Public understanding and use of antibiotics in England: findings from a household survey in 2017. BMJ Open 9:e030845. 10.1136/bmjopen-2019-030845 31662380 10.1136/bmjopen-2019-030845 PMC6830627

[31] Fal AM, Stelzmüller I, Kardos P et al (2024) Antibiotics Usage and Avoidance in Germany and Poland: Attitudes and Knowledge of Patients, Physicians, and Pharmacists. Antibiotics 13:1188. 10.3390/antibiotics13121188 39766578 10.3390/antibiotics13121188 PMC11672592

[32] WHO (2015) WHO multi-country survey reveals widespread public misunderstanding about antibiotic resistance. https://www.who.int/news/item/16-11-2015-who-multi-country-survey-reveals-widespread-public-misunderstanding-about-antibiotic-resistance

[33] Khatri J, Shakya R, Shrestha R, Shrestha S (2025) Disposal of unused and expired medications: A study of knowledge, attitudes, and practices among community pharmacy visitors. SAGE Open Med 13:20503121251375355. 10.1177/20503121251375355 41018130 10.1177/20503121251375355 PMC12464398

[34] Theodoropoulou A, Lisi M, Rolision J, Sirota M (2025) The effects of communicating illness diagnostic and treatment information and C‑reactive protein test results on people’s antibiotic expectations. Br J Health Psychol 30:e70020. 10.1111/bjhp.70020 40891384 10.1111/bjhp.70020 PMC12403044

[35] Broniatowski DA, Klein EY, May L et al (2018) Patients’ and Clinicians’ Perceptions of Antibiotic Prescribing for Upper Respiratory Infections in the Acute Care Setting. Med Decis Making 38:547–561. 10.1177/0272989X18770664 29847253 10.1177/0272989X18770664 PMC6274591

[36] Thorpe A, Lee RA, Fagerlin A et al (2025) Characteristics and Antibiotic Preferences of US Adults Reporting Frequent Use vs No Use of Antibiotics. JAMA Netw Open 8:e251429. 10.1001/jamanetworkopen.2025.1429 40116831 10.1001/jamanetworkopen.2025.1429 PMC11929022

[37] Sievert EDC, Korn L, Gross M et al (2024) Communicating diagnostic uncertainty reduces expectations of receiving antibiotics: Two online experiments with hypothetical patients. Appl Psychol Health Well-Being 16:1459–1478. 10.1111/aphw.12536 38500005 10.1111/aphw.12536

[38] Smith MJ, Wattles BA (2022) Appropriate Antibiotic Prescribing—The Safer and Less Expensive Choice. JAMA Netw Open 5:e2214160. 10.1001/jamanetworkopen.2022.14160 35616946 10.1001/jamanetworkopen.2022.14160

[39] Sievert EDC, Korn L, Gross R et al (2025) Recovery attributions and future expectations for antibiotics after precautionary prescribing. Eur J Public Health 35:1020–1025. 10.1093/eurpub/ckaf146 40857357 10.1093/eurpub/ckaf146 PMC12529262

[40] Thorpe A, Sirota M, Orbell S, Juanchich M (2021) Effect of information on reducing inappropriate expectations and requests for antibiotics. Br J Psychol 112:804–827. 10.1111/bjop.12494 33543779 10.1111/bjop.12494

[41] O’Donoghue T, Rabin M (1999) Doing It Now or Later. Am Econ Rev 89:103–124. 10.1257/aer.89.1.103

[42] Böhm R, Holtmann-Klenner C, Korn L et al (2022) Behavioral determinants of antibiotic resistance: The role of social information. Appl Psychol Health Well-Being 14:757–775. 10.1111/aphw.12345 35103398 10.1111/aphw.12345 PMC9544926

[43] Santana AP, Korn L, Betsch C, Böhm R (2023) Promoting prosociality toward future generations in antibiotic intake. J Health Psychol 28:1024–1037. 10.1177/13591053221149526 36721947 10.1177/13591053221149526

[44] Plechatá A, Makransky G, Böhm R (2024) A randomized controlled trial investigating experiential virtual reality communication on prudent antibiotic use. Npj Digit Med 7:244. 10.1038/s41746-024-01240-3 39266716 10.1038/s41746-024-01240-3 PMC11392957

[45] Sirota M, Juanchich M (2024) Seeing an apocalyptic post-antibiotic future lowers antibiotics expectations and requests. Commun Med 4:141. 10.1038/s43856-024-00567-y 38997505 10.1038/s43856-024-00567-y PMC11245540

[46] Thorpe A, Sirota M, Juanchich M, Orbell S (2020) Action bias in the public’s clinically inappropriate expectations for antibiotics. J Exp Psychol Appl 26:422–431. 10.1037/xap0000269 32271052 10.1037/xap0000269

[47] Santana AP, Korn L, Betsch C et al (2024) Understanding and reducing inappropriate antibiotic use in the context of delayed prescriptions. Health Psychol 43:194–202. 10.1037/hea0001323 37870788 10.1037/hea0001323

[48] World Health Organization European Region Europe (2022) AMS Posters for GPs and Patients.

[49] Sievert EDC, Gross R, Korn L et al. (2025) Using a decision support tool to reduce inappropriate antibiotic use after a delayed prescription. Unpubliziertes Manuskript. 10.1111/aphw.70198 PMC13451548 42568140

